# EEG biomarkers for the diagnosis and treatment of infantile spasms

**DOI:** 10.3389/fneur.2022.960454

**Published:** 2022-07-28

**Authors:** Blanca Romero Milà, Kavyakantha Remakanthakurup Sindhu, John R. Mytinger, Daniel W. Shrey, Beth A. Lopour

**Affiliations:** ^1^Department of Biomedical Engineering, University of California, Irvine, Irvine, CA, United States; ^2^Department of Electronics and Biomedical Engineering, Universitat de Barcelona, Barcelona, Spain; ^3^Division of Pediatric Neurology, Department of Pediatrics, Nationwide Children's Hospital, The Ohio State University, Columbus, OH, United States; ^4^Division of Neurology, Children's Hospital Orange County, Orange, CA, United States; ^5^Department of Pediatrics, University of California, Irvine, Irvine, CA, United States

**Keywords:** epilepsy, functional connectivity, entropy, hypsarrhythmia, high frequency oscillation (HFO), long-range temporal correlation (LRTC), Detrended Fluctuation Analysis (DFA), phase amplitude coupling

## Abstract

Early diagnosis and treatment are critical for young children with infantile spasms (IS), as this maximizes the possibility of the best possible child-specific outcome. However, there are major barriers to achieving this, including high rates of misdiagnosis or failure to recognize the seizures, medication failure, and relapse. There are currently no validated tools to aid clinicians in assessing objective diagnostic criteria, predicting or measuring medication response, or predicting the likelihood of relapse. However, the pivotal role of EEG in the clinical management of IS has prompted many recent studies of potential EEG biomarkers of the disease. These include both visual EEG biomarkers based on human visual interpretation of the EEG and computational EEG biomarkers in which computers calculate quantitative features of the EEG. Here, we review the literature on both types of biomarkers, organized based on the application (diagnosis, treatment response, prediction, etc.). Visual biomarkers include the assessment of hypsarrhythmia, epileptiform discharges, fast oscillations, and the Burden of AmplitudeS and Epileptiform Discharges (BASED) score. Computational markers include EEG amplitude and power spectrum, entropy, functional connectivity, high frequency oscillations (HFOs), long-range temporal correlations, and phase-amplitude coupling. We also introduce each of the computational measures and provide representative examples. Finally, we highlight remaining gaps in the literature, describe practical guidelines for future biomarker discovery and validation studies, and discuss remaining roadblocks to clinical implementation, with the goal of facilitating future work in this critical area.

## Introduction

Infantile spasms (IS) is a seizure type typified by brief muscle contractions, often occurring in clusters, with the peak incidence at 4–7 months of age (1). Recently, the syndrome associated with IS has been re-named “infantile epileptic spasms syndrome” (IESS) by the International League Against Epilepsy (2). While IESS represents only a fraction of all pediatric epilepsies, the consequences of the disease are among the most severe (Table 1). IESS is associated with high rates of mortality and morbidity, lifelong refractory seizures, and extraordinary health care costs. In children with IESS, the ongoing epileptic activity contributes to severe cognitive and behavioral disabilities associated with a progressive cognitive decline (14, 15). IS was strongly associated with a developmental and epileptic encephalopathy called West syndrome – classically defined as the combination of IS, developmental slowing or regression, and an electroencephalography (EEG) pattern known as hypsarrhythmia (16). 50–70% of children with IS will exhibit other seizure types and 18–50% will later develop Lennox Gastaut Syndrome (17).

**Table 1:** Compared to all pediatric epilepsies, infantile epileptic spasms syndrome is rare, but it has high rates of refractory seizures, developmental delay, mortality, and treatment costs.

	**Infantile spasms**	**All pediatric epilepsy**
Incidence	2–5/10,000 (1)	50–100/10,000 (3)
Refractory seizures	64% (4)	33% (5)
Development. delay	88% (6), (7)	51% (8)
Mortality	13% before age 2, (7) 10% before age 3 (4) 9% before age 9 (9)	2–12 per 1,000 person-years (10), (11)
Cost	>$200 k, first 12 months (12)	$2–5 k/year (13)

One major challenge in achieving good outcomes for children with IS is the need for early diagnosis and treatment. A short lag time between IS onset and treatment is a favorable prognostic factor, with delays as short as a few weeks being detrimental (18). Moreover, the risk of developmental delay increases when the characteristic EEG findings associated with IS are present for longer than 3 weeks (19). Unfortunately, effective treatment can be delayed by failed recognition and misdiagnosis (20). In 38% of IS cases, proper diagnosis is not given at the first physician visit (21); most commonly, no specific diagnosis is given, or the child is misdiagnosed with gastroesophageal reflux (20, 21). Approximately 30% of children are diagnosed more than 1 week after IS onset, partially due to poor awareness of IS among healthcare providers (22).

Outcomes in IESS are also negatively affected by medication failure and IS relapse. A positive, short-term treatment response is defined by the cessation of IS and resolution of hypsarrhythmia when present (23). Roughly 40–45% of children will not respond to an initial standard therapy (6, 24), defined as ACTH, prednisolone, or vigabatrin (24). Of those that do initially respond, 20–33% will have a relapse of IS (24, 25). In the case of ACTH therapy, the time to relapse can be anywhere from 5 days to 25 months after the completion of treatment (26, 27). To date, there are no methods for robustly predicting long-term, sustained response or relapse.

Given these major challenges, tools to assess objective diagnostic criteria, predict initial medication response, and measure the likelihood of relapse would help clinicians make critical management decisions. Given the importance of the EEG evaluation in the diagnosis and treatment of IS, such as the identification of hypsarrhythmia, the development of EEG biomarkers has been a promising avenue of research to address this need. Here, we review the current state-of-the-art in EEG biomarkers for IS, which generally fall into two categories: (1) *Visual EEG biomarkers*, which are based on human identification and characterization of EEG signal patterns and features, and (2) *Computational EEG biomarkers*, in which computers are used to calculate quantitative features of the EEG, such as power, symmetry, and functional connectivity networks. Both categories are discussed, with each section organized based on the biomarker application (diagnosis, treatment response, prediction, etc.). This highlights the potential value of such biomarkers and the current state of the field, as well as identifying key areas where additional research is needed.

## Visual inspection

### Diagnosis

Clinically, the diagnosis of IS relies on the clinical history, with the use of home-video of typical events being particularly helpful, and EEG inspection to look for evidence to support the diagnosis of epileptic encephalopathy (22, 28). Hypsarrhythmia is defined as high-voltage, asynchronous slow waves associated with focal or multifocal spikes that can vary in duration and location (15, 27). This section aims to highlight visual inspection-based studies that present alternatives to traditional diagnosis methods.

#### Hypsarrhythmia

While the presence of hypsarrhythmia on the EEG was historically perceived to be important for the diagnosis of West syndrome, many children with new onset IS will not have this classic pattern. Indeed, not long after the term “hypsarythmia” was coined by Gibbs and Gibbs (16), several authors noted the absence of this pattern in many children with IS (29). Multiple contemporary single center cohorts have confirmed that only about 60% of children presenting with IS have hypsarrhythmia (30, 31). In addition, despite readers' high confidence in their assessment, the determination of hypsarrhythmia has poor inter-rater reliability (IRR) (32). These shortcomings of hypsarrhythmia highlight the need for a quantifiable, reliable, and unbiased method of interictal EEG analysis for children with IS.

#### BASED score

**Figure 1:**
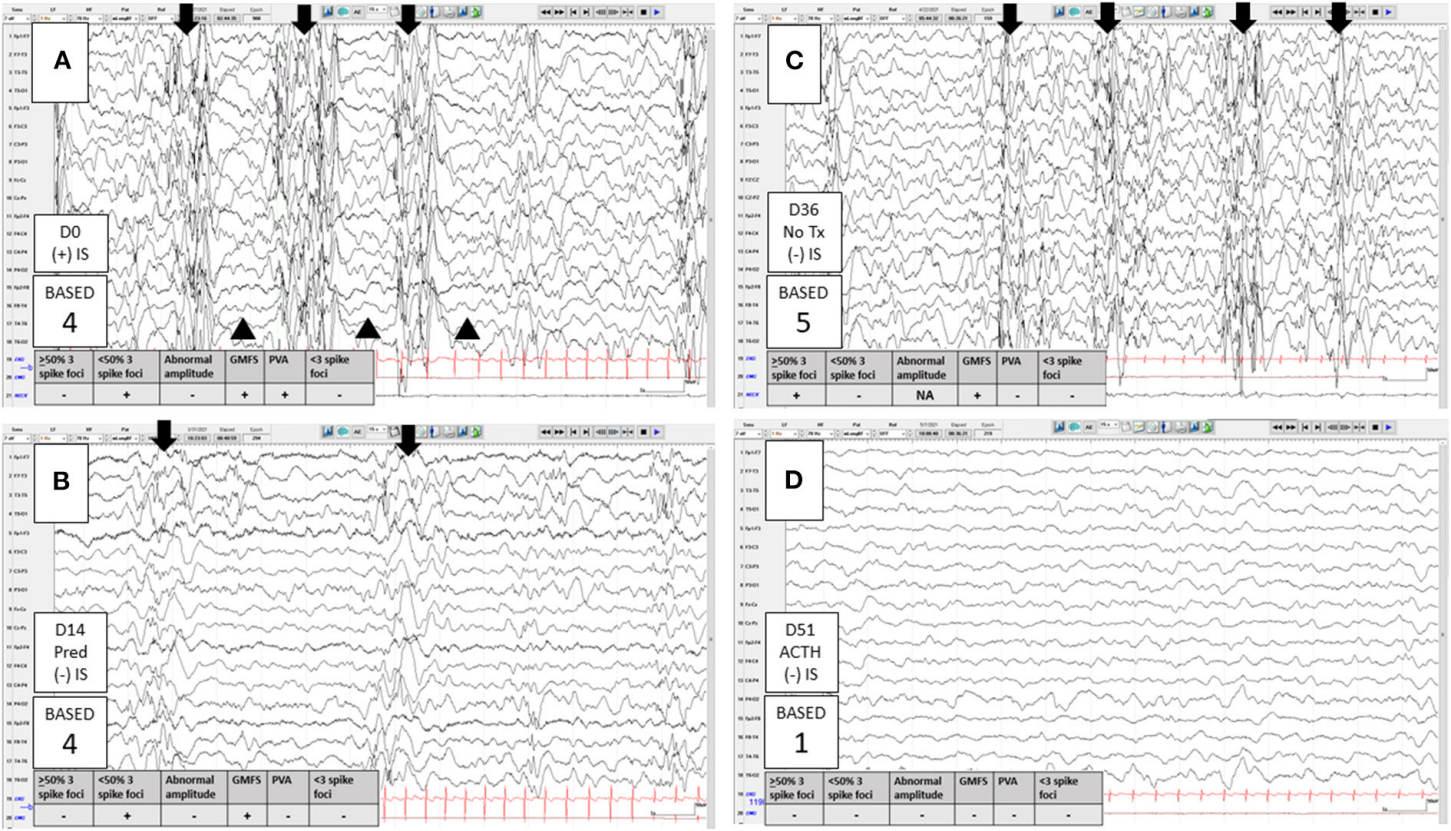
Clinical application of the 2021 Burden of AmplitudeS and Epileptiform Discharges (BASED) score. A 4-month-old infant born at 39 weeks gestational age, with known developmental delay due to hypoxic ischemic encephalopathy, presented with new onset infantile spasms (IS). Onset of IS to treatment initiation was 1 day. **(A)** Day 0 (diagnosis and treatment), just <50% of 1 s (s) bins within a 5-min (m) epoch of sleep included at least one spike and there were grouped multifocal spikes (GMFS, arrows) as well as paroxysmal voltage attenuations (PVA, arrowheads). The presence of either GMFS or PVA indicated a BASED score of 4 (EEG findings to suggest a probable epileptic encephalopathy [EE]). **(B)** Day 14, IS and PVA resolved with high-dose prednisolone, with a subjective reduction in the burden of spikes. However, because of persistent GMFS (now less well-formed), the BASED score remained 4. At this point, there was no electrographic remission (pretreatment scores of 4 or 5 must improve to a 3 or less for remission). With the resolution of IS and subjective improvement on the EEG, the treating clinician did not pursue additional treatment at that time (despite the lack of electrographic remission by BASED score criteria). **(C)** Day 36, still no IS but the EEG showed a higher burden of spikes, now with >50% of 1-s bins having at least one spike in a 5-m epoch of sleep, indicating a BASED score of 5 (EEG findings to suggest a definite EE). Better formed GMFS (arrows) were present. Notice that the background wave amplitude assessment is not reliable when the burden of spikes is ≥50%. High-dose adrenocorticotropic hormone (ACTH) was started at that time. **(D)** Day 51, all spikes resolved with ACTH. The background was slow and disorganized indicating a BASED score of 1 (Any definite non-epileptiform abnormality). This score, with the persistent remission of IS, indicated electro clinical remission. Longitudinal bipolar montage, Sensitivity 10 mv/mm, LFF 1 Hz, HFF 70 Hz, 15 s per page. D, days; NA, not applicable; Pred, high-dose prednisolone.

The Burden of AmplitudeS and Epileptiform Discharges (BASED) score, initially reported in 2015 (33) and revised in 2021 (34), is an interictal EEG grading scale for children with IS. This scoring method is centered on the assessment of background wave amplitude and the overall burden of epileptiform discharges (spikes). Important features of the score include grouped multifocal spikes, paroxysmal voltage attenuations, and percentage of 1-s bins that include at least one spike in 5 min of sleep (Figure 1). The scale ranges from 0 (normal) to 5 (most epileptic) (Table 2). All elements of the BASED score have moderate to high levels of IRR and have high correlation with the presence or absence of IS (34). The latter finding suggests that the assessment of BASED score elements are useful for the identification and diagnosis of children with IS. Seeing these interictal features in an infant should prompt the EEG reader to carefully review the EEG for IS, extending the study if needed. In addition, when there is interictal EEG evidence to suggest an epileptic encephalopathy by BASED score criteria, the diagnosis of IS can be made if there are typical clustering events by *either* caregiver's report or home-video (34). This approach allows for a diagnosis without the need to capture the ictal manifestation of IS using an extended video-EEG.

**Table 2:** Description of BASED scores using the 2021 criteria.

**2021 BASED Score**	**Description**
0	Normal
1	Any definite non-epileptiform abnormality
2	<3 spike foci AND no channel with abnormal high amplitude
3	>3 spike foci <50% of one second bins AND no channel with abnormal high amplitude, OR <3 spike foci but >1 channel with abnormal high-amplitude
4 (Probable EE)	>3 spike foci <50% of one second bins AND >1 channel with abnormal high amplitude, OR Not meeting criteria for 5 but includes GMFS or paroxysmal voltage attenuations
5 (Definite EE)	>3 spike foci that are >50% of one second bins

*BASED: Burden of AmplitudeS and Epileptiform Discharges, GMFS: grouped multifocal spikes, EE: epileptic encephalopathy. Apply score 3–5 to the most epileptic 5-minute epoch of sleep; if no score reached, apply score 0–2 to the remainder of the study. Adapted from Mytinger et al. (34). See this reference for complete BASED score rules and definitions*.

#### Fast oscillations

One study visually analyzed paroxysmal fast activity (PFA) as a candidate EEG biomarker of epilepsy (35). PFA consisted of transient EEG events in the beta or gamma frequency bands with a duration ranging from 200 ms to 8 s, typically occurring from 1 to >100 times in a 20-min interictal non-REM sleep epoch. This marker had low sensitivity, as PFA was found in only 28% of children with seizures; however, when present, it identified children with epilepsy with a 97% accuracy. In that study, 11 out of 13 children with IS exhibited PFA.

### Treatment response

Treatment response is classified as a binary *all-or-none* outcome, with the goal of treatment being electro clinical remission, i.e., the complete resolution of clinical IS and lack of any EEG findings to suggest an ongoing epileptic encephalopathy (23). The historical standard for the determination of electrographic remission has been the resolution of hypsarrhythmia. However, for the significant number of children without hypsarrhythmia on presentation, this methodology is inadequate (28). The 2021 BASED score provides an alternative method for the assessment of remission in children with IS.

#### BASED score

In a retrospective consecutive sample EEG study, the post-treatment BASED score (measured at a median of about 1 month following treatment) correlated well-with the presence or absence of IS. In that study, electrographic remission was defined as pretreatment scores of 4 (EEG findings to suggest a probable EE) or 5 (EEG findings to suggest a definite EE) improving to ≤ 3, and pretreatment scores of 3 improving to ≤ 2 (34).

### Outcome prediction

Currently, there are no clinically validated tools to predict the long-term response to treatment or the relapse of IS. However, the following studies evaluated candidate predictive biomarkers based on visual EEG analysis.

#### EEG patterns

As noted, hypsarrhythmia is the classic EEG pattern associated with IS. It was hypothesized that hypsarrhythmia may disturb physiological EEG patterns such as sleep spindles, which are thought to positively impact childhood development (36). Despite this, no correlation was found between the cessation of hypsarrhythmia or recurrence of sleep spindles with long-term outcomes in children with an unknown etiology and normal development prior to IS onset (34, 36). Furthermore, symmetric sleep spindles did not distinguish pretreatment and post-treatment studies in children with IS (34). In fact, a majority (about 55%) of children with new onset IS had symmetric sleep spindles at diagnosis, suggesting that this sleep finding is a robust thalamocortical projection that may not be useful in the EEG evaluation of children with IS (34).

In a prospective observational study, the presence of hypsarrhythmia on the diagnostic EEG did not predict the response to treatment. In this study, treatment with standard therapy had the greatest impact on the likelihood of treatment response (24, 37–39). Children with IS who did not have hypsarrhythmia on the diagnostic EEG were less likely to receive standard therapy and thus were less likely to achieve remission (39). Other studies showed that children with epileptiform discharges on EEG after successful ACTH therapy had a higher probability of relapse than those with normalized EEGs, with a relapse occurring an average of 6.6 months after completing therapy (25, 27). However, even with optimal treatment, it may not be possible to normalize the EEG for many children with IS.

#### BASED score

One study suggested that the BASED score can predict relapse after an initial response with ACTH therapy. Children with positive treatment outcomes and a BASED score >3 (which may indicate that the epileptiform activity was not sufficiently and effectively controlled) had a higher risk of relapse within 1 year (26).

## Computational analysis

### Diagnosis

Many computational analysis techniques have been applied to EEG data with the goal of objectively identifying children with IS. Here we discuss each study in detail; they are also summarized in Table 3, along with studies of computational EEG biomarkers for assessment of treatment response and prediction of response and relapse.

**Table 3:** Summary of all studies of computational EEG biomarkers related to IESS.

**Biomarker type**	**Aim**	**Comparison population**	**Accuracy measure**	**Refs**.
Amplitude	Diagnosis	Healthy children	Statistical test	(40, 41)
Spectral power	Diagnosis	Healthy children	Statistical test, except in (42)	(40–42)
	Assess treatment response	Responders vs. non-responders	Statistical test	(43)
Entropy	Diagnosis	Healthy children	Statistical test	(41, 44)
	Assess treatment response	Responders vs. non-responders; healthy children	Statistical test	(44)
	Predict response	Responders vs. non-responders	Statistical test	(44)
Functional connectivity	Diagnosis	Healthy children	Statistical test	(41, 42, 45, 46)
		Drug-resistant focal epilepsies	Statistical test	(47, 48)
		Children with TSC who did not progress to IESS	Statistical test	(49)
	Assess treatment response	Responders vs. non-responders; healthy children in ref (46)	Statistical test	(43, 46)
	Predict response and relapse	Responders vs. non-responders; healthy children	Statistical test; AUC	(46)
		Sustained response vs. relapse	Statistical test	(43)
Fast oscillations and high	Diagnosis	Healthy children	Statistical test	(50–53)
frequency oscillations		Compared fast oscillations associated vs. unassociated with epileptiform discharges	Statistical test	(54)
		Ohtahara syndrome	None	(55)
	Assess treatment response	Pre-treatment vs. post-treatment; seizure free vs. not seizure free	Statistical test	(56)
		Pre-treatment vs post-treatment; most include comparison to healthy children	Statistical test	(50–54)
	Predict response and relapse	Sustained response vs. relapse	Statistical test	(56)
		Responders vs. non-responders	Statistical test	(51, 57)
Long-range temporal	Diagnosis	Healthy children	Statistical test	(41, 58)
correlations	Assess treatment response	Responders vs. non-responders; healthy children	Statistical test	(58, 59)
Phase-amplitude coupling	Diagnosis	Healthy children	Statistical test; AUC in (60)	(53, 60)
		Focal epilepsy; also affected vs. unaffected hemispheres in both groups	Statistical test	(48)
	Assess treatment response	Responders vs. non-responders	Statistical test	(57)
Combination of multiple metrics	Diagnosis	Healthy children	Cross-validation, sensitivity, specificity, AUC	(41, 53)
	Predict response and relapse	Sustained responders, non-responders, response with relapse	Statistical test	(43)

#### Amplitude and spectral power

Amplitude is the voltage range of an EEG signal, most commonly measured from broadband data that has been filtered in a similar manner as clinical EEG studies (e.g., 1–70 Hz). Spectral power is related to the magnitude of the EEG in distinct frequencies or frequency bands, which can be obtained by decomposing the signal using a technique such as the Fourier transform. During interictal periods, children with IS had significantly higher EEG amplitude and higher spectral power in all standard frequency bands compared to healthy controls (40–42). Two studies suggested that this may have been specific to EEG with hypsarrhythmia (40, 42). However, one study of 40 children with IS, of whom about two-thirds had refractory IS and only eight had hypsarrhythmia, found significantly higher EEG amplitude and power compared to approximately age-matched healthy controls (41). This feature of the EEG was present during both wakefulness and sleep (41).

#### Entropy

**Figure 2:**
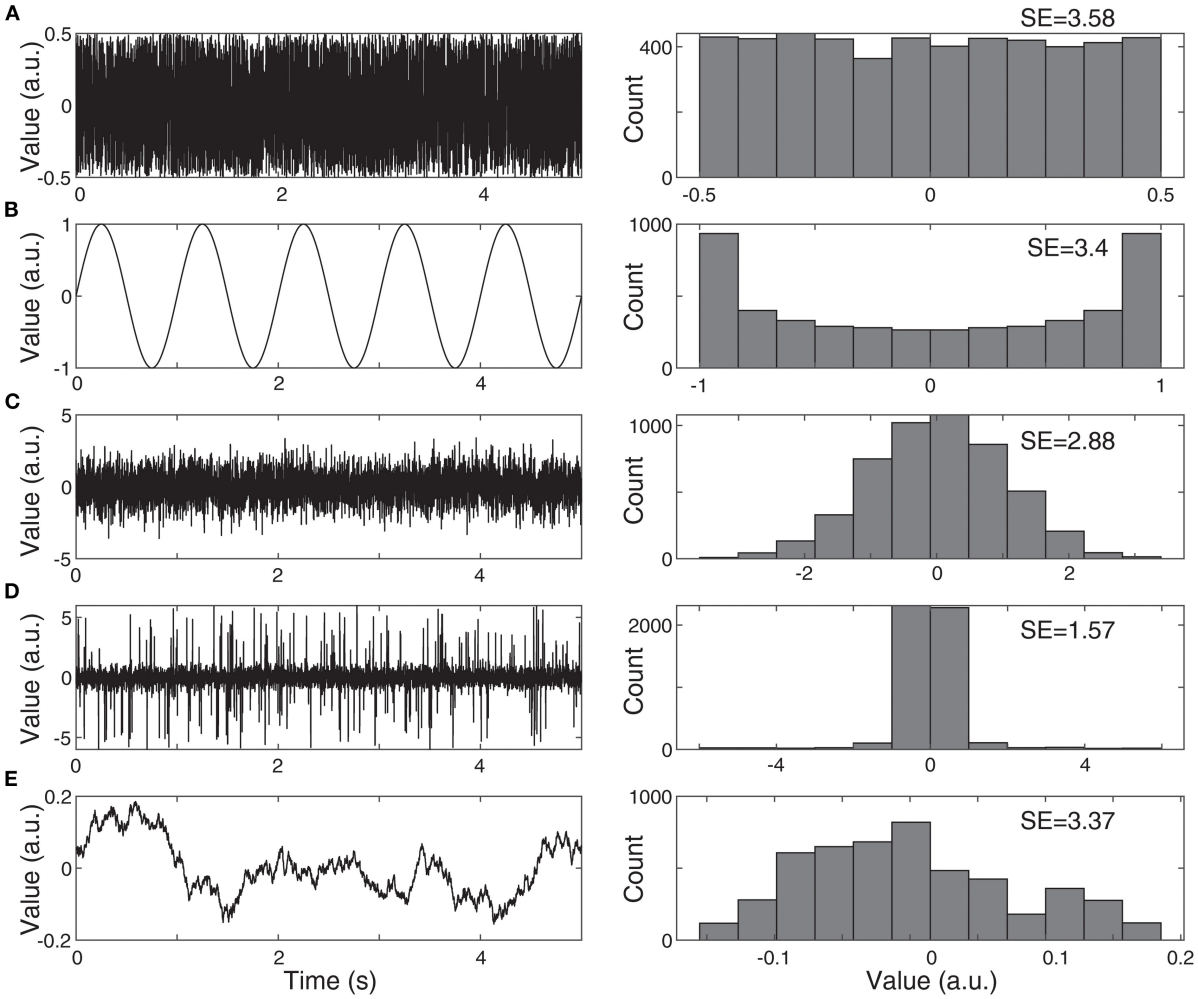
Examples of Shannon entropy (SE). **(A)** The SE of white noise is high, as all values occur with equal probability. **(B)** The SE of a sine wave is almost as high as white noise, despite the drastically different visual appearance. The SE is calculated based only on the signal's histogram, and both a sine wave and white noise have fairly flat histograms, with almost equal probability for all values. **(C)** Normally-distributed noise has lower SE, as it is more likely to have values near zero. **(D)** Normally-distributed noise with outliers has even lower SE, as the probability of having values near zero is increased, relative to the range of possible values. **(E)** Noise with a 1/f power spectrum, similar to EEG, generally has relatively high values of SE.

Entropy is a measure of the predictability of EEG data, with higher levels of uncertainty being associated with higher entropy. Shannon entropy is calculated using a histogram of EEG voltages during a specified window of time, and therefore all information about the temporal ordering of the data points is removed (Figure 2). In contrast, permutation entropy measures the relative occurrence of short patterns of data points, e.g., a set of three EEG data points can be categorized into one of six different sequences, depending on their relative values. If all sequences are equally likely to occur, indicating high uncertainty, the permutation entropy is high. In one study of children with IS, the EEG had lower Shannon entropy in the theta, alpha, and beta bands during sleep compared to healthy controls; compared to sleep, the results during wakefulness were more varied, with children with IS demonstrating higher entropy in the delta band, but lower entropy in the alpha band (41). The permutation entropy was also generally lower in infants with IS, particularly in the delta and theta bands during wakefulness and the delta and beta bands during sleep (41). Another entropy measure, called multiscale entropy, suggested that the total EEG complexity in IS patients was significantly lower than in healthy children (44).

#### Functional connectivity networks

**Figure 3:**
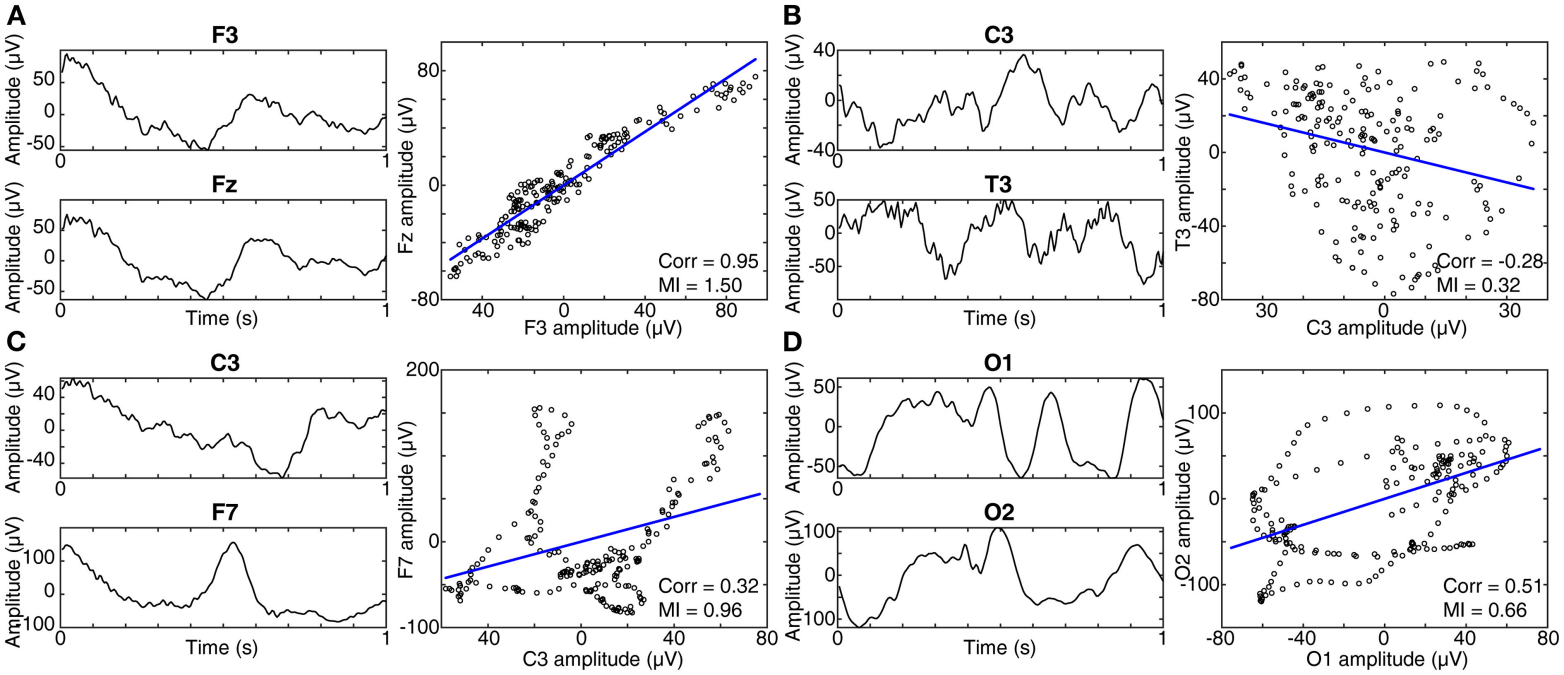
Linear vs. non-linear connectivity measures. **(A)** EEG signals recorded concurrently from two electrodes, F3 and Fz, are shown on the left. In the panel on the right, each data point represents one time point from the EEG signals, with the value of F3 on the horizontal axis, and the value of Fz on the vertical axis. In this case, the signals exhibit a linear relationship and thus have high values of both correlation and mutual information (MI). Recall that correlation is sensitive only to linear relationships, while MI is sensitive to both linear and non-linear interactions. **(B)** When the two EEG signals are unrelated, no trend can be seen in the right panel, and the values of correlation and MI are low. **(C)** When the relationship between the signals is non-linear, the MI is high, while the correlation is low. **(D)** A second example of a non-linear relationship, with medium correlation and MI.

Measurements of functional connectivity assess statistical relationships between EEG signals recorded from two or more spatial locations, with the goal of estimating an underlying functional connectivity network (FCN) (61). In the case of *bivariate* functional connectivity, these relationships are assessed independently between pairs of EEG electrodes. There are many techniques for measuring functional connectivity, and they can be broadly categorized based on whether they are sensitive to linear and/or non-linear interactions in the data (Figure 3).

Cross-correlation and coherence are linear, bivariate functional connectivity measurements that have been applied to IS. Cross-correlation measures the correlation between two signals as they are shifted in time relative to one another; when used to measure connectivity, the correlation with no time shift (also called zero time lag) can be excluded, as such synchronous signals with high correlation may be the result of volume conduction, rather than true connectivity (62). Significant correlations at non-zero lags, typically measured up to a maximum lag of 200 ms, are thought to indicate related signals shifted in time due to trans-synaptic connectivity. This technique is typically applied to a broadband EEG signal. In multiple studies, children with IS exhibited stronger cross-correlation FCNs compared with healthy controls (41, 45, 46). This held true when the FCNs were measured in both wakefulness and sleep; however, FCNs measured during sleep had stronger connections than those measured during wakefulness, for both children with IS and healthy controls (41, 63). These cross-correlation networks were found to be individualized, rather than stereotyped within a subject group, (64), and they had high test-retest reliability, with stable networks produced from as little as 150 s of EEG data (45, 46, 63).

In contrast to cross-correlation, coherence (which is closely related to magnitude-squared coherence) is typically applied to EEG data that have been filtered within a narrow frequency band, and it is therefore a frequency-specific measurement of functional connectivity. Coherence is sensitive to both the EEG power and the phase difference between the two signals, with high coherence occurring when the two signals exhibit a consistent phase difference during periods of high power. During sleep, children with IS and hypsarrhythmia exhibited higher coherence than controls in the delta, theta, alpha, and beta frequency bands, particularly for long-distance connections (42). Short-range connections in the frontal lobe were weaker than controls in the theta and beta frequency bands (42). In another study, source localization was applied to the EEG of children with IS and hypsarrhythmia, and coherence was calculated between a reference source and the rest of the brain (47). In all children, the reference region, defined as the source with the highest delta power, was located in the occipital cortex, and significant coherence was found with both cortical and subcortical regions. Then results from a linear, directed measure of connectivity called renormalized partial directed coherence (65) suggested that the brainstem was driving the activity in the putamen and cerebral cortex (47).

Non-linear connectivity metrics can uncover more complex relationships between pairs of EEG signals, and some metrics are sensitive to both linear and non-linear interactions (Figure 3). Mutual information measures the shared information between two signals and is equal to the entropy of signal one, plus the entropy of signal two, minus the joint entropy. Linear and/or non-linear relationships between signals can result in a high value of mutual information. Mutual information values were found to be higher in infants with tuberous sclerosis complex (TSC) that would later go on to develop IS, in comparison to those with TSC that did not develop IS (49). This appeared to be a relatively global change, occurring between most EEG channel pairs and in all frequency bands.

While the mutual information is a function only of the distribution of EEG values, which ignores the temporal ordering of the data points, many non-linear functional connectivity measures rely on measurements of relative phase as a function of time. Mean phase coherence measures the variance of the phase difference between two signals during a window of time; a tightly clustered distribution of phase differences, indicating a consistent phase difference over time, will result in a high value. This metric is not a function of the EEG amplitude or power. A study of three infants exhibiting IS and hypsarrhythmia found that hypsarrhythmia was associated with higher mean phase coherence than ictal electrodecremental events, contrary to the notion of hypsarrhythmia as a “chaotic” pattern (66). The phase lag index is also calculated using the phase difference between two signals, with the highest values occurring when the phase differences are either consistently positive or consistently negative. Signals with a distribution of phase differences centered around zero have a low phase lag index; this reduces the impact of volume conduction and common sources on the connectivity measurement. The phase lag index also revealed significantly stronger connectivity in children with IS, compared to healthy controls, in the delta band in both wakefulness and sleep (41). Lastly, the synchronization likelihood is a non-linear measure of functional connectivity that can be calculated at each point in time to quantify the dynamics of coupling between two channels. It is sensitive to both linear and non-linear interdependencies. This method relies on “embedding” each EEG time series in a higher dimensional state prior to comparing the activity in two independent channels, which means that high synchronization likelihood can occur even if the two EEG time series do not have a similar visual appearance (67). In children with IS and hypsarrhythmia secondary to perinatal arterial ischemic stroke, synchronization likelihood suggested stronger interhemispheric connectivity, as well as stronger intra-hemispheric connectivity in the affected hemisphere (48). This high connectivity was found in almost all frequency bands (ranging from 0.5 Hz to 70 Hz), and the authors speculated that this was related to the generation and modulation of hypsarrhythmia (48).

#### Fast oscillations and high frequency oscillations

**Figure 4:**
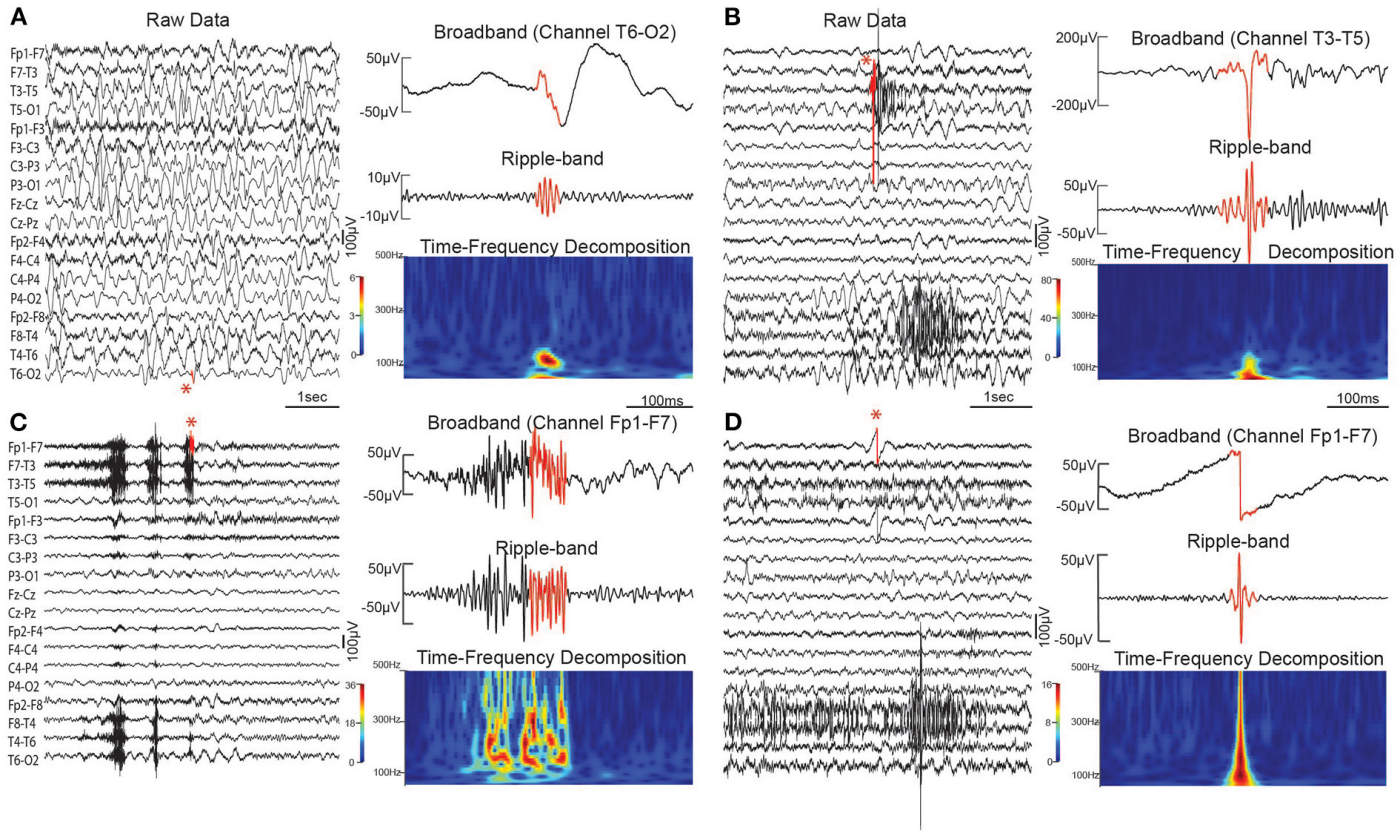
Scalp HFO and artifact examples. **(A)** Scalp HFO in bipolar channel T6–O2 in a child with IS. **(B)** Sharp artifact in channel T3–T5. **(C)** Muscle artifact in Channel Fp1–F7. **(D)** DC shift artifact in Channel Fp1–F7. Sharp artifacts, muscle activity, and DC shifts can all cause false positive HFO detections. In each subfigure, 5 s of broadband EEG is shown (left), with the red portion of the signal indicating the detected event (also marked by a red asterisk). On the right is the broadband EEG, with the detected event in red (top), the bandpass filtered EEG in the ripple frequency band (middle), and the time-frequency decomposition for the same segment of EEG (bottom).

Many studies over the past two decades have demonstrated a link between epilepsy and the occurrence of HFOs in EEG (68). These transient, electrographic events are typically defined as four or more oscillations at a high frequency (>80 Hz) that are distinct from the background (Figure 4). While the precise frequency ranges vary from study to study, HFOs are often categorized as ripples (80–250Hz) and fast ripples (250–500 Hz). The term fast oscillation (FO) can refer to ripples or oscillations in the gamma (40–80Hz) frequency band (50, 55). Both HFOs and FOs, measured with scalp EEG, have shown promise as biomarkers for the diagnosis of IS. While fully automated detection of HFOs and FOs is becoming more prevalent (69), most studies that rely on visual detection of high frequency events also incorporate computational techniques such as time-frequency analysis or quantitative analysis of event features. Therefore, we discuss all such studies here, regardless of their specific method for event detection.

During ictal periods, scalp EEG measurements have been used to demonstrate the occurrence of high frequency events in the beta (70), gamma (70–74), and ripple frequency bands (74). At the onset of spasms, focal beta and gamma activity were found to occur prior to motor symptoms; the majority of cases showed this increase in EEG power beginning in a single hemisphere, despite the fact that the EEG was often visually symmetric (70). In a separate study of four children, ictal ripple locations were concordant with lesions identified via neuroimaging (72). Temporally, gamma and ripple events were found to occur most frequently near the trough point of the ictal slow wave (74).

Many studies have documented the occurrence of scalp HFOs and FOs during interictal periods, as well. These were most frequently studied using slow-wave sleep EEG, with detection of events in the gamma and ripple frequency bands (50, 51, 54, 55), the ripple band (52), or the ripple and fast ripple bands (53). Scalp HFOs and FOs occurred more frequently in children with IS than in healthy controls (50, 52, 53) and more frequently during sleep than wakefulness in IS (52). The energy of gamma band events during sleep was also found to be higher in children with IS than healthy controls (51). Spatially, one study of IS reported that ripple rates were significantly higher than controls in the posterior parasagittal region and significantly lower in the frontal region (52), but a second study found the highest rates of gamma, ripple, and fast ripple events in the temporal and frontal lobes of children with IS (51).

#### Long-range temporal correlations

**Figure 5:**
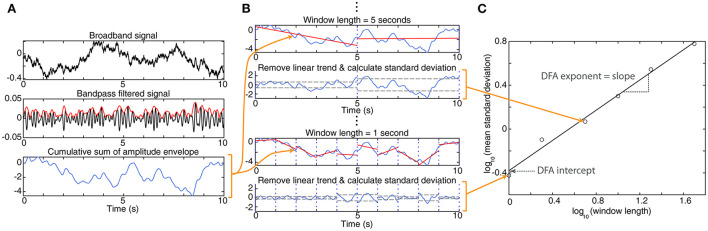
Detrended fluctuation analysis. **(A)** To start, the broadband EEG signal (top panel) is bandpass filtered (black line, middle panel), and the amplitude envelope is determined (red line). Then the cumulative sum of the amplitude envelope is calculated (bottom panel). The remaining calculations use this cumulative sum. **(B)** The cumulative sum is divided into windows of a fixed length; typically, these windows overlap by 50%, but no overlap is shown in this figure for clarity. Within each window, a linear trend is fit to the data (red line). Then, for each window, the linear trend is subtracted, and the standard deviation of the residual is calculated (gray dashed line). This process is repeated for windows of varying lengths. Here, we show examples of 5-s and 1-s windows. **(C)** For each window length, the mean standard deviation across all windows is calculated. This is plotted on a logarithmic scale vs. the window length. For EEG data, this will typically result in data points with a linear relationship. A linear trend line is fit to the data points on this graph; the slope of the line is the DFA exponent, and its intersection with the vertical axis is the DFA intercept.

Healthy human EEG, with its 1/f power spectrum, has a scale-free structure. This is conceptually similar to a fractal; no single time scale can be used to accurately characterize the dynamics present in EEG (75). Such systems also characteristically exhibit long-range temporal correlations, referring to the idea that the EEG amplitude fluctuations happening now are correlated to those happening in the future, at longer timescales than would be expected. Detrended Fluctuation Analysis (DFA) is one method for quantifying long-range temporal correlations, and thus the scaling behavior of a system (76) (Figure 5). This analysis results in two metrics that can be used for analysis: the DFA exponent (the slope of the line), and the DFA intercept (the intercept of the line with the vertical axis) (Figure 5C). In all frequency bands, children with IS and hypsarrhythmia exhibited lower DFA exponent values than healthy controls, indicating weaker long-range temporal correlations (58). In that study, children with IS but not hypsarrhythmia had DFA exponents that were not statistically different from controls. A separate study of 40 infants with IS (only eight of whom had hypsarrhythmia) also reported no difference in DFA exponent between cases and control subjects in most frequency bands during wakefulness and sleep (41). However, IESS was associated with higher DFA intercept values, compared to controls, in almost all frequency bands during wakefulness and sleep.

#### Phase-amplitude coupling

**Figure 6:**
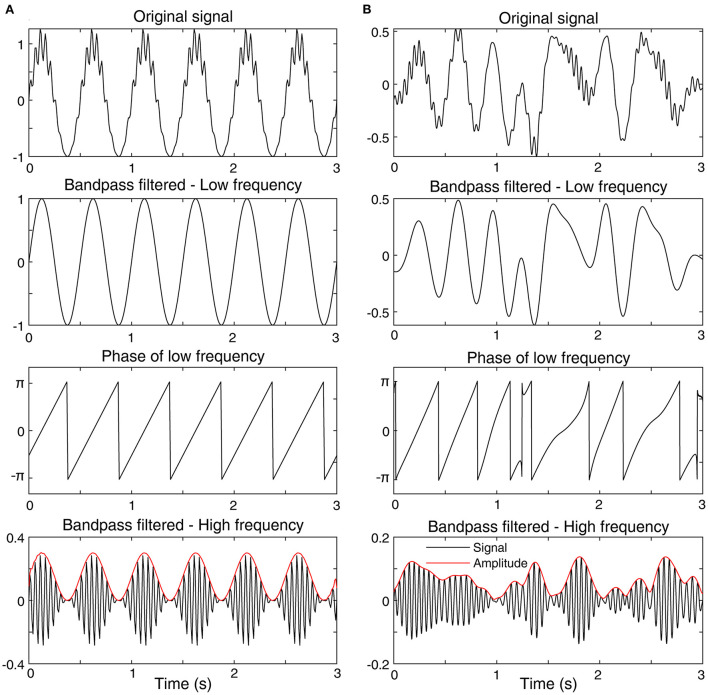
Examples of phase-amplitude coupling. **(A)** A signal with high phase-amplitude coupling. **(B)** A signal with low phase-amplitude coupling. Each subfigure shows the original signal (top), the bandpass filtered signal in a low frequency band (2nd from top), the phase of the low frequency bandpass filtered signal (3rd from top), and the bandpass filtered signal in a high frequency band (bottom). The amplitude envelope of the high frequency signal is also shown (bottom, red). In subfigure **(A)** the bursts of high amplitude, high frequency activity always occur at the peak of the low frequency signal, represented by a phase of zero. This consistent relationship will lead to a high value of phase-amplitude coupling. In contrast, in subfigure **(B)**, the bursts of high frequency activity occur randomly with respect to the low-frequency phase, which will result in a low value of phase-amplitude coupling.

Phase-amplitude coupling refers to a consistent relationship between the phase in one frequency band and the amplitude of a second (usually higher) frequency band, often within the same EEG channel (Figure 6). In IS, phase-amplitude coupling was assessed between the phase of the delta frequency band and the amplitude of the gamma frequency band using a metric called the modulation index. Children with active spasms had higher modulation index values based on the phase in 3–4 and 0.5–1 Hz bands and the amplitude in the HFO frequency band (80–500 Hz), compared to normal children who underwent overnight evaluation for suspected spasms (53). Then an automated process for EEG cleaning, preprocessing, and calculation of delta-gamma phase-amplitude coupling was developed and applied to 40 children with IS and 20 healthy controls (60). In that study, classification of cases and controls based on modulation index had an area under the curve (AUC) of 0.98 when using sleep EEG and an AUC of 0.85 during wakefulness. A separate study found that delta-gamma modulation index was significantly higher in the affected hemisphere for children with West Syndrome secondary to perinatal arterial ischemic stroke (48).

#### Classification using combined metrics

A logistic regression model was developed to classify children with and without active spasms using features based on HFO rate, the modulation index, and the presence of hypsarrhythmia. HFO rate and modulation index performed better than the metric based on hypsarrhythmia, and the overall accuracy on an independent dataset using cross-validation was 87.5% (53). A separate study used a multivariable logistic regression model to classify children with IS from approximately age-matched, healthy controls (41). The metric, obtained using a forward stepwise approach, was a linear combination of phase-lag index connectivity (delta frequency band), Shannon entropy (beta band), and DFA intercept (beta band), with all components measured during sleep. The classifier achieved an AUC of 0.96 on the age-matched cohort; including older subjects, particularly those older than 4 years, caused the performance of the model to suffer.

### Assessment of treatment response

The ideal outcome after successful treatment of IS is complete resolution of the clinical spasms and normalization of the EEG; however, this is not always obtainable. As noted, successful treatment response is currently considered to include both resolution of clinical spasms and no evidence of hypsarrhythmia on the EEG, without specification regarding whether the EEG normalizes or not. Recently, computational analysis has been used to create tools capable of achieving quantifiable and more reliable measurements of treatment success.

#### Functional connectivity

Cross-correlation, as a measurement of functional connectivity, has demonstrated utility as a quantitative marker of treatment response in children with IS who were treated with ACTH and/or vigabatrin as first-line therapy. In a study of 21 children with IS, decreased functional connectivity strength following first-line treatment occurred in 100% (eleven out of eleven) of IS subjects with successful treatment response (46). However, a decrease in functional connectivity strength following treatment was not found to be specific to successful treatment, as seven of ten non-responders in the same study also exhibited decreased functional connectivity strength following first-line treatment. Increased functional connectivity strength following first-line treatment was only seen in three of the ten non-responders and none of the responders.

#### Power and coherence

Delta band power and delta band coherence have both been shown to decrease to a greater degree following treatment in responders than in non-responders (43).

#### Long-range temporal correlations

A significant increase in LRTCs was noted in children with pretreatment hypsarrhythmia that resolved following therapy, whereas no significant changes were seen in children whose hypsarrhythmia persisted. These results were independent of whether or not spasms resolved. When assessing all criteria for response, rather than just hypsarrhythmia, responders showed a greater increase in strength of LRTCs following treatment than non-responders. After treatment, responders were not significantly different from controls, while non-responders had weaker LRTCs. Together, these findings suggest that LRTCs, represented by the DFA exponent, can be impacted by the presence or absence of hypsarrhythmia, but it is also affected by whether the clinical spasms resolve or not (58, 59).

#### High-frequency oscillations and fast oscillations

Rates of high frequency events decreased in the majority of children with IS after ACTH treatment, suggesting that HFOs may be a marker of disease burden in IS (50, 52). A study of children with TSC and West Syndrome found that FOs associated with spikes significantly increased with the onset of West Syndrome and decreased after ACTH treatment, while no such significant results were found for FOs occurring independently of spikes (54). Further, children with favorable treatment response exhibited consistent reductions in HFO rate during sleep when measured several days or more after treatment initiation, whereas non-responders demonstrated inconsistent changes (increases, decreases, and no changes) in HFO rate following treatment (53). McCrimmon et al. demonstrated that these post-treatment changes in HFO rate can be detected during sleep as early as 1–2 days following initiation of ACTH treatment, however, no significant changes in HFO rate were seen during wakefulness at this interval (52). Among IS treatment responders, no significant changes in average HFO energy were seen after treatment (51). Following ACTH treatment, the number of ripples and number of ripple channels were significantly lower in responders than non-responders; however, no difference in spectral power of ripples was noted between the two groups. Additionally, following ACTH therapy, the decrease in ripple number, ripple spectral power, and ripple channels for each patient was significantly greater for responders than non-responders (56).

#### Entropy

Total EEG complexity was used to evaluate EEG signals of children with IS who did not respond to non-ACTH anti-seizure medications and were subsequently treated with ACTH. Total EEG complexity was measured before and after subsequent ACTH treatment, as well as after 6 months of follow-up. The total EEG complexity following 14-days of ACTH therapy was not significantly different between ACTH responders and non-responders; however, at 6 months of follow-up, ACTH responders exhibited higher total EEG complexity than ACTH non-responders (44).

### Prediction of treatment response and relapse

In clinical practice, no EEG-based tools are currently used to predict treatment response or spasms relapse. Therefore, this section summarizes studies that evaluated EEG characteristics as potential predictive biomarkers of sustained treatment response or relapse.

#### Functional connectivity

In a study with children treated with ACTH and/or vigabatrin, strong pretreatment functional connectivity was associated with favorable short-term treatment response (46).

#### Power and coherence

Delta power and delta coherence measured in children with IS before and after ACTH treatment showed that decreases in these measurements following treatment were greater in responders who did not subsequently relapse compared to non-responders and responders who relapsed (43).

#### High frequency oscillations

In a study utilizing ACTH for treatment of IS, no differences were seen in the pretreatment EEGs of 17 responders and 22 non-responders across multiple HFO measurements, including (1) number of ripples, (2) spectral power of ripples, and (3) number of ripple channels. However, among the 17 responders, when measured post-treatment, all three of these measurements were significantly lower for those that did not relapse within 6 months of treatment (*n* = 12), compared those who relapsed (*n* = 5). The decrease in ripple number, ripple spectral power, and ripple channels for each patient following treatment was also significantly greater in the non-relapse group than in the relapse group (56). A separate study showed no differences between responders (*n* = 6) and non-responders (*n* = 7) in pretreatment HFO modulation index at different slow-wave activity (SWA) passbands; however, group wise differences in the phase coupling angle distributions were reported (57). Lastly, the pretreatment average HFO energy during sleep has shown to be lower for responders than non-responders (51).

#### Entropy

Pretreatment total EEG complexity was higher among children with IS who responded to non-ACTH anti-seizure medications, compared to children with IS who did not respond to non-ACTH anti-seizure medications. However, total EEG complexity, measured after failure of non-ACTH anti-seizure medications, but before initiation of ACTH treatment, was not significantly different between 13 ACTH responders and nine ACTH non-responders (44).

## Discussion

We reviewed the visual and computational EEG-based biomarkers that have been studied in association with the diagnosis, treatment response, and outcome prediction of children with infantile spasms (IS). The motivation behind most of these studies has stemmed from the major challenges associated with enacting effective treatment for IS, including the risk of delayed or erroneous diagnosis and the high rates of initial treatment failure and relapse. Many of the previously referenced works also highlight other barriers associated with the management and study of IS.

The first of these barriers is that IS is a relatively uncommon disease, affecting 2–5 out of every 10,000 live births (77). This results in limited sample sizes for single-center and small multi-center studies and makes biomarker validation studies on large, blinded datasets logistically challenging. The challenges of biomarker research in this rare disease are further compounded by the second such barrier – the etiological heterogeneity of the disease, with hundreds of etiologies leading to the final common pathway of IS (78, 79). This heterogeneity undoubtedly contributes to the clinical inconsistencies previously described. Thus, discovering, validating, and clinically implementing objective biomarkers that reliably improve the outcomes of children with IS is paramount.

### Diagnosis

A correct and early diagnosis of IS enables timely prescription of standard therapy, which is associated with greater likelihood of successful treatment and the best possible child-specific outcome (39). Therefore, it is essential to rely on EEG analysis techniques that ensure a reliable diagnosis of IS.

To date, clinicians have universally relied on visual EEG analysis for the diagnosis of IS. The presence of hypsarrhythmia interictally is highly suggestive of an IS diagnosis, however, the absence of hypsarrhythmia does not rule out IS, as EEGs of up to 40% of children with IS will not exhibit hypsarrhythmia at the time of diagnosis (30, 31). Further, visual evaluation and analysis of the components of hypsarrhythmia exhibit poor IRR (32). Since 2015, the visually determined interictal EEG grading scale known as the BASED score has gained favorability as an adjunctive metric. When compared to the low IRR of hypsarrhythmia, the BASED score has high levels of IRR. The diagnosis of IS may be aided by the recognition of BASED score elements on the interictal EEG. For example, the presence of grouped multifocal spikes in sleep should prompt the EEG reader to carefully review the study for IS. In addition to the BASED score, PFA has been suggested as a visual EEG biomarker of IS, as PFA has been shown to indicate a high risk for epilepsy when detected in EEG signals of children. However, PFA is not consistently present in children with IS and is also not specific to IS (35).

Several quantitative EEG biomarkers, such as amplitude, spectral power, entropy, functional connectivity, HFOs, and long-range temporal correlations, have been studied in children with IS. Significantly higher amplitude and spectral power across all standard frequency bands was seen in the EEGs of children with IS, and was shown to consistently correlate with the presence of hypsarrhythmia across multiple studies (40–42). Some studies also found that elevated amplitude and spectral power was also seen in children with IS whose EEGs did not meet criteria for hypsarrhythmia, suggesting that amplitude and spectral power are not merely a surrogate for the presence or absence of hypsarrhythmia, but may provide more gradation to the pretreatment EEG evaluation of children with IS (41).

EEG-based entropy was also shown to be significantly lower in children with IS compared to healthy controls (41). This is consistent with what has been reported among other types of epilepsy (80–83). This finding, at first glance, appears in contradiction to the historical description of hypsarrhythmia as a visually “chaotic” pattern, as a high degree of chaos would suggest similarly high levels of entropy, or unpredictability of the EEG signal over time. However, recall that measures such as Shannon entropy do not incorporate the temporal ordering of data points into their calculation, so it is difficult to estimate entropy based on the visual appearance of the signals (Figure 2).

Multiple studies have shown that FCNs are consistently stronger in children with IS than in healthy controls during wakefulness and sleep and across multiple frequency bands, with higher levels of connectivity seen during sleep than wakefulness. This has been validated using linear and non-linear metrics (41, 45, 46). These network structures are highly child-specific. Studies have also shown that the presence or absence of hypsarrhythmia does not independently drive connectivity strength, as EEG recordings with high BASED scores are associated with a wide range of FCN strengths (46). This is in contrast with long-range temporal correlations, which have been shown to be consistently weaker than healthy controls in children with IS who have hypsarrhythmia, but were not significantly different than controls who had IS without hypsarrhythmia (58). Further, the presence of interictal epileptiform discharges in the EEGs of children with IS has been shown to be associated with increased FCN strength, however, models simulating epileptiform activity within normal infant EEG recordings suggest that this is most likely due to the underlying pathological network as opposed to spurious connectivity caused by the presence of a spiky waveform in the EEG (84).

Several studies have shown a strong association between scalp HFOs and IS during both ictal and interictal periods, suggesting high potential for use in a diagnostic biomarker. HFOs may also have value for localizing epileptogenic activity associated with IS (55, 70, 72). Most of these studies analyzed events in the gamma and ripple frequency bands, although one showed an association with fast ripples, as well (52, 53). While these events have a low amplitude when measured non-invasively with scalp EEG, and EEG artifacts such as muscle activity are a significant confounding factor, the repeated reports of significant differences between children with IS and controls suggests that they can be reliably measured. However, most studies have relied on visual marking or visual validation of automatically detected events; this provides maximal ability to reject false positive events based on the myriad of factors that must be considered, but it is subject to reviewer bias and is very time consuming. One study reported fully automated detection of ripples and found significant differences in HFO rate between children with IS and a control group of infants under evaluation for suspected IS who were later found to be neurologically normal (52). More work is needed on the development of robust methods for automated detection of scalp HFOs, with special consideration for unique EEG features associated with IS, such as hypsarrhythmia. In addition, while one study analyzed HFOs in all children suspected of IS (comparing those that were ultimately diagnosed to those that did not have active IS) (53), more systematic studies are needed to compare IS directly to other similar seizure types and syndromes, rather than healthy controls.

Phase-amplitude coupling has also shown promise as a classifier for children with IS when compared with healthy infants. Children with active IS have higher modulation index values based on the phase of the 3–4 and 0.5–1 Hz bands coupled with the amplitude of the HFO band (80–500 Hz) than healthy infants. Following an automated pre-processing algorithm, delta-gamma modulation index was able to classify children with IS from healthy controls with an AUC of 0.98 during sleep and 0.85 during wakefulness, demonstrating a very high level of accuracy. The ability to perform this classification based on awake EEG makes modulation index feasible for diagnostic use based routine EEGs which nearly invariably capture wakefulness during the 30–60 min of standard recording (53, 60).

Many of the computational metrics have shown to correlate well-with clinicians' diagnostic assessments or provide ancillary data to inform the diagnostic process. Given the complementary nature of many of these EEG characteristics, a multivariate approach to IS classification is logical. Multivariate models for IESS have recently emerged, including clinical and computational EEG metrics. These innovative techniques have demonstrated favorable IS classification with AUCs ranging from 0.80 to 0.98 (41, 53). As further complementary biomarkers for IS emerge, accuracy will likely improve.

Ideally, a standardized, multivariate tool that can be broadly implemented to aid in IS diagnosis would hasten the time from spasm onset to diagnosis and therefore improve the likelihood of successful treatment and optimize long-term outcome. Further, this tool may also yield utility in predicting the onset of IS in high-risk individuals, such as children with perinatal brain injury or tuberous sclerosis complex, if utilized with surveillance EEG recordings prior to IS onset.

### Treatment response

Treatment response describes the changes in a patient's condition after treatment. Determining the effects of a therapy on IS is essential to guide future management of the disease. Further, it is essential to consider that IS treatment response is likely not binary (responder vs. non-responder), in contrast to how IS treatment response has been historically and is currently categorized. In all likelihood, this binary classification of treatment response is due, at least in part, to the historical lack of objective, reliable, graded metrics of disease burden for IS. Standardizing quantifiable metrics of treatment response for IS and universally applying them would create significant benefits for IS management and research. For instance, if a clinical treatment resulted in a measurable partial treatment response but not complete response, this would suggest that continuing the treatment while adding a second agent would be the ideal course of action. Additionally, reliable graded metrics of treatment response would allow clinical trials to assess the efficacy of novel treatments with smaller sample sizes, which is paramount in a rare disease like IS. Several clinical and computational EEG-based biomarkers have shown promise for assessing treatment response in children with IS.

The BASED score has been presented as an alternative to hypsarrhythmia for evaluating treatment response in children with IS. As previously mentioned, the BASED score has demonstrated superior IRR when compared with clinician evaluation of hypsarrhythmia (34). Further, the BASED score allows for individualized quantification of EEG abnormalities in children with IS. This is integral for assessing treatment response, as quantifying improvement in the EEG following treatment initiation is essential, especially in children with IS who do not exhibit hypsarrhythmia on their pretreatment EEGs. This concept of individualized treatment response quantification using the BASED score was shown to be feasible, and treatment response was defined differently based on the pretreatment BASED score. For pretreatment BASED scores of 4 or 5, electrographic remission was defined as improvement to BASED score ≤ 3, whereas in children with pretreatment scores of 3, electrographic remission required post-treatment scores of ≤ 2 (34).

Computational techniques like functional connectivity, power, coherence, and HFOs have been tested as potential markers of treatment outcome, showing differentiative traits between responders and non-responders. In one study, FCN strength, measured with cross-correlation, decreased consistently following standard IS treatment in all responders, however, 70% of non-responders also demonstrated decreased FCN strength following treatment. In the same study, 30% of non-responders exhibited increased FCN strength following treatment – a finding that was not observed in responders (46). This suggests that a post-treatment decrease in FCN strength is a sensitive but not specific biomarker of favorable treatment response, while an increase in FCN strength is a specific but not sensitive biomarker of treatment non-response. Delta power and coherence showed similar trends, however, the degree of decrease following treatment in both metrics was found to be greater in responders as opposed to non-responders, group wise (43). Changes in DFA following first-line treatment closely mirrors whether the patient's post-treatment EEG met criteria for hypsarrhythmia or not (58). This suggests that DFA may function as an objective surrogate measure for hypsarrhythmia, though the sample size of this study was small. Further, the magnitude of DFA change was related to whether the clinical spasms resolved or not, suggesting that other factors beyond hypsarrhythmia may also affect long-range temporal correlations.

Changes in HFOs following treatment of IS are similar to those seen with FCNs. HFO rates during sleep consistently decrease following successful treatment of IS, however, rates may increase, decrease, or remain unchanged following unsuccessful treatment (53). Other studies reported similar decreases in HFO rate following successful IS treatment (54, 56), with evidence that these decreases may begin to manifest within 24–48 h of treatment initiation (52). Average HFO energy seems unaffected by treatment response (51). Overall, an increase in HFO rate following treatment is highly suggestive of treatment non-response, whereas virtually all responders will exhibit a decrease in HFO rate to some degree. This mirrors FCN changes with treatment, as previously described.

Multiscale entropy has not been broadly studied in IS, however, one study found no significant differences between responders and non-responders following treatment initiation. Nevertheless, 6 months later, ACTH responders exhibited higher total EEG complexity than non-responders (44).

Many of the above EEG metrics show promise for achieving quantifiable and reliable measurements of treatment success, however, large-scale work remains necessary to validate these potentially reliable and objective measures of treatment response.

### Outcome prediction

Treatment response prediction can objectively guide clinicians when making medical decisions about therapy. Stratification of children with IS by likelihood of treatment response would allow for more aggressive measures to be taken in those with higher risk of non-response or relapse, and conversely also allow less aggressive, less invasive treatments to be used in lower risk cohorts. Predicting the likelihood of relapse may also guide monitoring following successful treatment (e.g., a child with a high likelihood for relapse would likely require more frequent clinic visits and EEGs than one at low risk for relapse).

#### Prediction of initial treatment response using pretreatment EEG

Clinically, the presence of hypsarrhythmia prior to treatment initiation was not a predictor of treatment response. However, children without pretreatment hypsarrhythmia were less likely to receive standard therapy – an unsupported approach that decreased likelihood of treatment response (39). Using cross-correlation, very strong pretreatment FCNs were found to be associated with favorable treatment response in a small subset of children with IS treated with ACTH and/or vigabatrin (46). HFOs have shown mixed results as predictive biomarkers. HFO rates, spectral power, and channels, as well as HFO modulation index in slow-wave passbands, measured in pretreatment EEGs, have not demonstrated a robust ability to predict responders and non-responders (57). However, the average HFO energy during sleep was shown to be lower for responders than non-responders (51).

#### Prediction of subsequent relapse in treatment responders

The BASED score has shown promise for predicting relapse. Among treatment responders, those with post-treatment BASED scores of 3 or higher showed a high rate (89%) of subsequent relapse (26). Similarly, smaller decreases in delta power and delta coherence following treatment among responders have been shown to indicate higher risk of relapse. HFO rates, spectral power, and number of HFO channels were significantly lower among responders who did not relapse compared to those who subsequently relapsed (56).

### Gaps in knowledge and future directions

Much of the work to date studying EEG-based biomarkers of IS has utilized relatively small sample sizes and has been carried out at single centers or small groups of centers. Small studies typically are limited in the generalizability of their results and the likelihood of finding statistically significant conclusions, as well as the ability to study subsets of the population, i.e., focus on a specific IESS etiology. More recently, large multicenter efforts studying IS have proved fruitful, and further efforts to collect data from a large, diverse population of children with IS that can be shared and analyzed should be pursued. These types of studies present their own logistical barriers to implementation, such as adequate funding, data collection/storage, navigating the myriad of research regulations across different geographic regions, and standardizing data collection and treatment protocols.

Computational EEG biomarkers are becoming more commonplace in IS research, and their utility is often complementary to visual EEG analysis. Significant strides have been made to include more quantitative analysis within clinical EEG recording and interpretation. For example, software packages like *Persyst*® are designed to integrate into existing clinical EEG software, such as *NeuroWorkbench*® by *Nihon Kohden*. These software tools remain costly to implement and require expertise in computational EEG analysis to properly configure, and as of yet they are not universally adopted. Additionally, as most commercially available software uses proprietary algorithms, integrating customized analyses into the software is not easily done or, in many cases, is impossible. Therefore, universal integration of computational biomarkers into the standard clinical workflow for epilepsy practices remains a significant barrier to their widespread usage. This process can be facilitated by considering ease of implementation when choosing biomarker methodologies. Once computational analysis is fully integrated into EEG data collection and clinical review, a paradigm shift in clinical training would still be required for clinical epileptologists to be more broadly exposed to these techniques and their uses during their residencies and fellowships.

Finally, the discovery of biomarkers is only the first step on the road to their clinical acceptance and broad usage, as they must be rigorously validated. Biomarker discovery studies should be thoughtfully designed in ways that minimize bias and produce robust, reproducible results. Patient selection and EEG data segment selection should be randomized, multiple distinct EEG samples should be analyzed for each patient (to prove reliability of the measure), and states of consciousness should be considered. Further, the clinicians who clinically interpret the EEG studies and the researchers who perform the computational analysis of the EEG data should be blinded to treatment status and outcome (41). The tools to perform these analyses should be made freely and broadly available so research groups can validate each other's findings on independent datasets. Lastly, validation of biomarkers should be undertaken on large datasets, and validated biomarkers should then be tested in a clinical trial environment to ascertain their likelihood of impacting clinical practice.

It is not yet clear what final form the implementation of such a biomarker would take in clinical practice, as a fully validated biomarker does not yet exist. However, once found, it would ideally be seamlessly integrated into the clinical workflow using widely available software. The process of evaluating the biomarker would be automated such that accuracy can be guaranteed for a range of expertise levels. This would enable widespread adoption of the biomarker as an adjunctive tool for the diagnosis and management of IESS.

## Author contributions

BR has first authorship of the manuscript and performed data analysis, figure preparation, and literature search and selection, and also wrote the first manuscript draft and helped edit the manuscript. KR contributed data analysis and figure preparation. JM and DS contributed to figure preparation, provided clinical expertise, literature search and selection, and helped write and edit the manuscript. BL helped conceptualize and supervise the manuscript preparation, while performing data analysis, preparing figures, and writing and editing the manuscript. All authors contributed to the article and approved the submitted version.

## Funding

This work was supported by the UC Irvine California-Catalonia Engineering Program, through a Balsells Mobility Fellowship to BRM. Funding was also provided by the Children's Hospital of Orange County Physician Scientist Scholar's Program.

## Conflict of interest

The authors declare that the research was conducted in the absence of any commercial or financial relationships that could be construed as a potential conflict of interest.

## Publisher's note

All claims expressed in this article are solely those of the authors and do not necessarily represent those of their affiliated organizations, or those of the publisher, the editors and the reviewers. Any product that may be evaluated in this article, or claim that may be made by its manufacturer, is not guaranteed or endorsed by the publisher.
